# One Hidden Semantic Model Based on Intergroup Effects for E-Commerce

**DOI:** 10.1155/2022/7273728

**Published:** 2022-07-21

**Authors:** Yanli Li, Wensong Zhang

**Affiliations:** School of Economics and Management, Beijing Jiaotong University, China

## Abstract

E-commerce systems often collect data that clearly express user preferences without considering the remaining negative cases, which gives rise to the hidden semantic problem. In this paper, we improve the original hidden semantic model and propose an intergroup effect model that incorporates users' historical browsing behavior, user type, and browsing content; by adopting the weighting and add weighting factors, we can predict users' preferences for different products more accurately and match the candidate products with users' current behaviors, so as to give more reasonable and effective product recommendation results; by adding the group effect model of user group and product group, we can achieve more accurate prediction of user preferences and make the recommendation more reasonable and effective. The research shows that the hidden semantic method based on intergroup effects information is better than other basic methods at a certain identified evaluation stage. In practice, users' purchasing preferences change with time, and using a hidden semantic method based on intergroup effects recommendation can effectively improve the recommendation quality of e-commerce recommendation systems.

## 1. Introduction

E-commerce does not have the geographical restrictions that exist in traditional business models, and consumers have more room for choice. However, with the rapid expansion of information on the Internet [1, 2], the phenomenon of “information overload” has emerged, i.e., when users browse or buy on the website, there may be multiple categories for users to choose because the website is full of various items; users have an increasingly strong demand for information filtering; their tastes are becoming more and more personalized; and changes in user interests need to be urgently captured in a timely manner. The need for users to quickly find items they like from a vast amount of resources is a pressing issue, and users also need a technology that can automatically deliver items of interest based on their needs. From the retailer's perspective, while the opportunity to develop e-commerce is enormous, it must address a higher level of complexity. Namely, the increase in the number of users and products in recent years has required retailers to develop a systematic recommendation list. This list is either a list of items for targeted customers or a list of users for targeted items, making it easier for users to find information of interest and for information to be delivered more easily to its appropriate users. The method of generating a list of associations by explicitly expressing the user's interest or extracting potential information from the user's behavior is called hidden semantic.

Therefore, personalized recommendation technology has been rapidly developed and applied to e-commerce recommendation systems, which can help users to avoid getting lost in the huge amount of item information, help them to make decisions, select the desired items, and increase the sales volume of items [3, 4]. In recent years, along with the arrival of “big data era,” information overload, and information explosion, the hidden semantic technology in personalized recommendation technology has gradually become the most mature and successful technology so far [5–7]. However, in many cases, e-commerce systems often collect data that explicitly express user preferences, i.e., positive cases (explicit feedback), without considering the remaining negative cases, including implicit feedback and nonexistent feedback (data that cannot explicitly express user preferences or does not express user preferences), which is the problem of hidden semantic.

So, the gaps of the current studies are as follows: compared with hidden semantic, there are fewer studies on one hidden semantic, and the sparse data, fewer positive cases, and more confusing negative cases make the research difficult, but it also shows that one hidden semantic has potential research value and space. In this paper, based on a large amount of domestic and foreign related literature, we choose to propose solutions to the problems of data sparsity and intergroup effects of hidden semantic. The contributions of the work are as follows: (1) we summarize the basic methods researched in this field and then use singular value decomposition and matrix weighted approximation methods to design a hidden semantic recommendation algorithm and establish a corresponding model; (2) in the setting of the weights, based on previous researchers' studies, intergroup effects information is introduced: user's recent browsing information related to user relationship management and item input market information related to item life cycle; and (3) the designed algorithm is experimentally simulated and compared with the existing basic methods to verify its effectiveness. Regardless of the collaborative filtering system, its purpose is mainly to provide users with recommendation suggestions for similar items. The optimization formula mainly considers the case of only 1 in the joint matrix, which is imperfect for single-class collaborative filtering. In single-class collaborative filtering, weights can be set to indicate the importance of different preferences of potential users for various items or different data sets.

The organization of the paper is as follows. The second part is a literature review, the third part is the proposed model, the fourth part is the experimental design, the fifth part is the experimental results and discussion, and finally the last part is the study conclusion.

## 2. Related Works

In the hidden semantic problem, for a user, there can be explicit feedback (positive examples) indicating his behavior, such as movie ratings, item scores, and reviews, while apart from these positive examples, the remaining ones are negative examples, including two types of cases, implicit feedback and non-existent feedback. Implicit feedback, as opposed to explicit feedback, usually does not directly show a user's tendency, such as that user browsing a website, listening to a song, retweeting a tweet, or buying a product. It is usually unclear to us whether the user has seen the video or purchased the item. Not knowing does not mean that the user has not watched or purchased the item; perhaps the user has watched or purchased the item on another site, so there are no negative examples for the hidden semantic problem. The absence of negative examples does not mean that recommendations cannot be made. Domain-based algorithms, such as item-based hidden semantic models, can implement recommendations on data sets with only positive examples. This is because the basic idea is to draw a slightly larger circle than the positive examples outside the set of positive examples and then recommend those videos or items that are similar to the videos or items they have seen [3, 4], but the absence of negative examples means that the learning algorithm is basically impossible to implement due to the fact that learning algorithms mostly draw a surface between positive and negative examples, and if there are no negative examples, there is no surface.

The difficulty of recommendation based on implicit feedback is the lack of negative examples, i.e., the researcher can know explicitly what the user's interests are but not what the user dislikes. Pan and Scholz [5] defined such a problem as hidden semantic. Currently, the main idea to solve this difficulty is to construct negative examples, which can include the following three strategies:

1. All unselected or unrated items or items are treated as negative examples and certain weights are set. For example, Wang et al. [6] used Probabilistic Matrix Factorization (PMF) technique to treat click data as positive examples and all other data as negative examples; Hu et al. [7] and Steck [8] set a relatively small confidence level (weight) on the premise that all unselected or unrated items or items are treated as negative examples, a strategy intended to control the impact of the introduced uncertain negative examples with the help of this relatively small weight; Paterek [9] used Singular Value Decomposition (SVD) for this problem, while Rendle et al. [10] used KNN (K Nearest Neighbor) based hidden semantic model to solve this problem.
2. A certain sample of unselected or unrated items or items is taken as negative examples. For example, Chen et al. [11], Shi et al. [12], and others have made recommendations by assuming the randomly sampled items as negative examples. The idea of this type of strategy requires the assumption of a premise that the vast majority of items not selected by users are those that users will not choose. Rong et al. [13] have proposed a sampled binary SVD algorithm, which is based on a uniform sampling, a biased user sampling, biased item sampling, and so on, to extract a set similar in size to the positive examples from the missing values as negative examples; among the three sampling methods, the second one is the best, the first one is the second, and the third one is worse.
3. According to the specificity of the environment to which the recommendation is applied, certain rules are artificially set so as to specify negative examples, but this method especially relies on the special application environment as well as human a priori knowledge, so it is not generalizable. For example, Jiang et al. [14] studied the retweeting behavior of microblog users by first assuming that the behavior of unretweeted tweets in a valid online session is a negative example, which needs to be based on the prerequisite that users are likely to be online during this time and have viewed these tweets but not retweeted them.

The treatment of negative and unobserved data is the key to solve the hidden semantic problem. There is little research on this issue, and a large number of studies, including the abovementioned literature, are mainly concerned with weighting since weighted low-rank approximation can improve the recommendation effect of unobserved data. Most of the related studies in the field focused on factorization models based on latent factors with different weights proposed by Pan and Scholz [5] Sindhwani et al., Pan et al., Shen et al., Wang et al., Yin et al., Kang et al., Tian et al., and Shi et al. [15–22] simplified the weight setting scheme with a formula to propose another optimization variable that provides a measure of the treatment of unobserved data.

In the experimental process of constructing negative examples, these methods inevitably introduce noise since there is no guarantee that there are no potential positive examples among these introduced negative examples as seen in Table 1, i.e., items or items that the user will choose in the future. However, for traditional recommendation models, it is necessary to construct negative examples because this is the only way to exploit the “unselected” information. In essence, whether it is random sampling, setting weights, or setting artificial rules, a balance is sought between using “unselected” information and controlling the introduction of noise. In addition, constructing negative examples has the drawback that it increases the size of the data significantly and is therefore not suitable for large amounts of data.

## 3. Research Method

### 3.1. Basic Model

Collaborative filtering algorithms mainly include user-based collaborative filtering, item-based collaborative filtering, and hidden semantic models. Among them, user-based collaborative filtering and item-based collaborative filtering are analysed by statistical methods based on data analysis [23], so they are also called memory-based or neighborhood-based collaborative filtering. The implicit semantic model, on the other hand, is a model based on learning data through algorithms such as machine learning and makes predictions and recommendations based on the model, which belongs to model based collaborative filtering. In this paper, we choose to use the implicit semantic model for recommendation.

The hidden semantic model is one kind of collaborative filtering algorithm, which is a matrix decomposition model. The core idea of the Latent Semantic Model is to use the automatic clustering of user behavior statistics to discover the implicit features linking users' interests and items and to find potential classifications through these implicit features, so as to realize the connection establishment between users and products.

Due to the differences between individual users, there are bound to be some differences in preferences. Therefore, the user's preference should be judged based on this aspect. Therefore, the goal of recommendation is to recommend items under the category that the user is interested in. Moreover, if two items are preferred by multiple users at the same time, then there is a high probability that both items belong to the same category. The implicit semantic analysis is based on the user behavior statistics to get the classification and automatically calculate the weight of each category. The exact number of categories is decided by human, and the more categories there are, the finer the granularity of the classification. The idea is to decompose the user's historical behavior record matrix of items into a user-implied factor matrix and an item implied factor matrix and use the product of the implied factor matrix as the user's preference for items.

The hidden semantic model calculates the interest of user *u* in item *i* by using the formula as follows:

(1) $$\begin{array}{c} \text{Preference}\left(u,i\right)={\hat{r}}_{u,i}=p_{u}^{T}q_{i}=\sum_{k=1}^{K}p_{u,k}q_{i,k}, \end{array}$$

where *p*_*u*,*k*_ and *q*_*i*,*k*_ are both parameters of the model. *p*_*u*,*k*_ measures the relationship between user u's interest and the *k*th hidden class, and *q*_*i*,*k*_ measures the relationship between the *k*th hidden class and item *i*. *p*_*u*_ is the matrix of user-implied factors obtained after matrix decomposition, and *q*_*i*_ is the matrix of item-implied factors obtained after matrix decomposition. The two parameters calculated by learning the dataset, which contains items of ‘like' and ‘dislike'. In the recommendation system, the data of user behavior are divided into explicit feedback data and implicit feedback data; the explicit feedback data are mostly the rating data of users, but the implicit feedback data are more applied in practice; this dataset usually has no negative samples, only what items users like, which is positive samples.

In order to obtain the implied factor vectors *p*_*u*_ and *q*_*i*_ in the model, a loss function needs to be set and optimized to obtain the most appropriate *p*_*u*_ and *q*_*i*_. The loss function is shown as follows:

(2) $$\begin{array}{c} C=\sum {\left(r_{u,i}-{\hat{r}}_{u,i}\right)}^{2}+\;\lambda p_{u}^{2}+\lambda q_{i}^{2}, \end{array}$$

where *λp*_*u*_^2^+*λq*_*i*_^2^ is the regularization term to prevent overfitting and *λ* is obtained experimentally.

The stochastic gradient descent algorithm or alternating least squares method is generally used to achieve the minimization solution. The stochastic gradient descent algorithm is used to optimize the loss function by first taking partial derivatives of the above equation to obtain the following:

(3) $$\begin{array}{c} \frac{\partial L}{\partial p_{u,k}}=-2q_{i,k}+2\;\lambda p_{u,k},\\ \frac{\partial L}{\partial q_{i,k}}=-2p_{u,k}+2\lambda q_{i,k}. \end{array}$$

The parameters are then iteratively updated until the loss function converges, where *α* is the learning rate, and the larger *α* is, the faster the iterations fall.

(4) $$\begin{array}{c} p_{u,k}=p_{u,k}+\alpha \left(q_{i,k}-\lambda p_{u,k}\right),\\ q_{i,k}=q_{i,k}+\alpha \left(p_{u,k}-\lambda q_{i,k}\right). \end{array}$$

### 3.2. Improved Model

Regardless of the hidden semantic system, its purpose is mainly to provide users with recommendation suggestions for similar items. The above optimization formula mainly considers the case of only 1 in the joint matrix, which is imperfect for hidden semantic. In hidden semantic, weights can be set to indicate the importance of different preferences of potential users for various items or different data sets.

So, we summarize the basic methods researched in this field and then use singular value decomposition and matrix weighted approximation methods to design a hidden semantic recommendation algorithm and establish a corresponding model; and in the setting of the weights, based on previous researchers' studies, intergroup effects information is introduced: user's recent browsing information related to user relationship management and item input market information related to item life cycle; lastly, the designed algorithm is experimentally simulated and compared with the existing basic methods to verify its effectiveness.

Therefore, in this paper, we adopt the idea of weighting and add weighting factors to the optimization model after introducing the parameters above in an attempt to find a low-rank matrix *Y* that approximates *X* to the maximum extent.

(5) $$\begin{array}{c} \underset{U\geq 0,V\geq 0}{\text{argmin}}\lambda \left(U_{F}^{2}+V_{F}^{2}\right)+\sum WL\left(X,Y\right). \end{array}$$

The application of the weighting idea is set to have two extreme weights in the hidden semantic model to apply a weight-based low-rank matrix to approximate as follows.

Set the weights of all positive factors in the matrix to 1 and the weights of other factors to 0. Set *X*^1^ and *X*^0^ as follows:

(6) $$\begin{array}{c} X^{1}=\left\{\left(i,j\right):X_{i,j}=1\right\},\\ X^{0}=\left\{\left(i,j\right):X_{i,j}=0\right\}. \end{array}$$

 
*X*^1^ denotes all 1 (with transaction records) in matrix *X* corresponding to (*i*, *j*)
 
*X*^0^ denotes all 0 (no transaction records) in *X* corresponding to (*i*, *j*)

Based on the above weight settings, the optimization formula can be further modified as

(7) $$\begin{array}{c} \underset{U\geq 0,V\geq 0}{\text{argmin}}\lambda \left(U_{F}^{2}+V_{F}^{2}\right)+\sum_{i,j\in X^{1}}W_{i,j}L\left(X_{i,j},u_{i}^{T}v_{j}\right), \end{array}$$

where $W_{i,j}=\left[\begin{array}{cc} 1 & \forall \left(i,j\right)\in X^{1}\\ 0 & \forall \left(i,j\right)\in X^{0} \end{array}\right]$.

However, the model ignores the nonpurchasing group, and in order to meet the requirement of setting different weight values for different types of factors in hidden semantic, the formula (7) can be further modified as follows:

(8) $$\begin{array}{c} \underset{U\geq 0,V\geq 0}{\text{argmin}}\lambda \left(U_{F}^{2}+V_{F}^{2}\right)+\sum_{i,j\in X^{1}}W_{i,j}L\left(1,u_{i}^{T}v_{j}\right)+\sum_{i,j\in X^{0}}W_{i,j}L\left(0,u_{i}^{T}v_{j}\right). \end{array}$$

The formula includes all types of users: Better buyers, potential buyers, and nonbuyers through the setting of weight *W*_*i*,*j*_ values. After setting the corresponding weights *W*_*i*,*j*_, equation (8) can be rewritten as the following formula:

(9) $$\begin{array}{c} \underset{U\geq 0,V\geq 0}{\text{argmin}}\lambda \left(U_{F}^{2}+V_{F}^{2}\right)+\Omega ⊗{\left(X-UV\right)}_{F}^{2}, \end{array}$$

where $\Omega_{i,j}=\sqrt{W_{i,j}}$ and ⊗ denotes an operation by elements, meaning multiplying the corresponding elements inside two different matrices.

Finally, the final optimization scheme is obtained according to after multiplicative substitution as follows:

(10) $$\begin{array}{c} V=V⊗\frac{U^{T}\left(\Omega ⊗X\right)}{U^{T}\left(\Omega ⊗\left(UV\right)\right)+\lambda V},\\ U=U⊗\frac{\left(\Omega ⊗X\right)V^{T}}{\left(\Omega ⊗\left(UV\right)\right)V^{T}+\lambda U}. \end{array}$$

### 3.3. Algorithm Design

Compared with alternating least squares, a more efficient parallel algorithm is proposed to estimate the implied factor vector, which embeds the inverse fitting algorithm into alternating least squares; it can avoid a large number of matrix operations and large memory storage, achieve scalable computation in practice, improve the training efficiency of the model, and save training time. The parallel computation process of the optimization method is as follows:

① Initialization: set the initial values of (*P*^(0)^, *Q*^(0)^, *S*^ (0)^, *T*^(0)^) and the tuning parameter *λ*
② Item implied factor: Iteratively estimate  *Q*^(*l*)^ and *T*^(*l*)^
(i) *Q*^(*l*)^ ← *Q*^(*l* − 1)^, *T*^(*l*)^ ← *T*^(*l* − 1)^
(ii) For each item with *i* = 1,…, *m*, use equation to calculate *q*_*i*_^(*l*)new^
(iii) For each item with  *J*_*j*_, *j* = 1,…, *M*, use equation to calculate  *t*_*j*_^(*l*)new^
(iv) Stop iteration if 1/*mKQ*^(*l*)new^ − *Q*_*F*_^(*l*)2^+1/*MKT*^(*l*)new^ − *T*_*F*_^(*l*)2^ < 10^−5^, or *Q*^(*l*)^ ← *Q*^(*l*)new^, *T*^(*l*)^ ← *T*^(*l*)new^, and return to step 2(ii)
③ Item implied factors: Iteratively estimate *P*^(*l*)^和*S*^(*l*)^
(i) Let *P*^(*l*)^ ← *P*^(*l* − 1)^, *S*^(*l*)^ ← *S*^(*l* − 1)^
(ii) For each user with *u*=1,2,…, *n*, use equation to compute *p*_*u*_^(*l*)new^
(iii) For each user group  *V*_*v*_, *v*=1,…, *N*, use equation to calculate  *s*_*v*_^(*l*)new^
(iv) Stop iteration if  1/*nKP*^(*l*)new^ − *P*_*F*_^(*l*)2^+1/*nKS*^(*l*)new^ − *S*_*F*_^(*l*)2^ < 10^−5^, otherwise assign *P*^(*l*)^ ← *P*^(*l*)new^, *S*^(*l*)^ ← *S*^(*l*)new^, and return to step 3(ii)
④ Stopping criteria: Stop if  1/*nKP*^(*l*)^+*S*_*c*_^(*l*)^ − *P*^(*l* − 1)^ − *S*_*c*,*F*_^(*l* − 1)1^ + 1/*mKQ*^(*l*)^+*T*_*c*_^(*l*)^ − *Q*^(*l* − 1)^ − *T*_*cF*_^(*l* − 1)2^ < 10^−3^ otherwise make  *l* ← *l*+1 go back to step 2

## 4. Experimental Design

### 4.1. Algorithm Design

For the user clustering subsystem, Web mining technology can be used to extract the implicit rating information of users on item categories from user log files and user purchase databases, establish the user-item category rating matrix, and then generate recommendation results for users according to the implementation method of hidden semantic technology.

In this paper, we use the personalized recommendation subsystem to optimize the model recommendation results by using personalized recommendation techniques.

(1) Establish user-item rating matrix.
In the actual research, the items on the website can be categorized by concept hierarchy, and the user's browsing and purchasing situation of a certain type of item can be calculated from the server logs and sales records so that different weights can be assigned to the user's browsing and purchasing items, and finally the user's rating value of a certain type of item can be calculated by the formula to establish the user rating matrix.
In this paper, we choose to apply the method based on singular value decomposition to reduce the dimensionality of the initial matrix to get a matrix with a smaller dimensionality to facilitate the operation.
(2) Select the blank items to be rated and predict the unrated items according to different ways of setting the weights.
(3) Calculate the similarity between users.
The main choice is the relevant similarity metric formula (described accordingly in Chapter 2) to calculate the similarity between users.
(4) Find the set of neighbors that are similar to the target and use the following formula to make predictions:
  (11) $$\begin{array}{c} P_{u,i}=\bar{Y_{u}}+\frac{\sum_{v\in N}\text{sim}\left(u,v\right)\times \left(Y_{v,i}-\bar{Y_{v}}\right)*\Delta }{\sum_{v\in N}\left(\left|\text{sim}\left(u,v\right)\right|\right)*\Delta }, \end{array}$$
where different expressions are chosen according to different methods, mainly applied in intergroup effects user-centric weighting methods and intergroup effects item-centric weighting methods.
(5) Generate the corresponding recommendation list.
(6) Perform relevant validity analysis to verify the proposed model.

### 4.2. Dataset Selection

The dataset chosen for this experiment is the MovieLens dataset, which was collected by the GroupLens Research project group at the University of Minnesota Movie listings. Currently, the Website has more than 70,000 users and over 5,000 movies with ratings. The movie ratings are integers from 1 to 5, and the higher the value, the more users like the movie, and the movies that are not rated account for most of the overall data, which reflects the data sparsity problem mentioned above.

For all the ratings, this paper intercepts a part of the MovieLens dataset provided by the GroupLens research group, which was collected from September 1997 to April 1998 and contains 943 users and 1682 movies, in which each user has rated at least 20 movies, with a total of 100,000 rating records. This data set is very sparse, with a sparsity rating of 1,100,000/(943*∗*1682) = 6.305%, i.e., only 6.305% of the items have ratings. In the specific experiments later, 80% of this part of the data is selected as the training set, and the remaining 20% of the data is the test set. The data in the training set are used for training, and the recommendations are tested with the data in the test set to obtain the relevant values, which are then analysed to determine the strengths and weaknesses of the recommendation algorithm.

In the database of this dataset, there are six data tables: users, movies, ratings, age, genres, and occupation. The recommended movies mainly include the following categories: action, adventure, animation, children's, comedy, crime, documentary, drama, fiction, fantasy, film-noir, horror, musical, mystery, romance, sci-fi, thriller, war, and Western.

### 4.3. Evaluation Criteria for Recommendation Quality

For hidden semantic recommendations, the prediction evaluation criteria commonly used by numerous researchers are divided into the following two main types.

#### 4.3.1. Statistical Accuracy Criterion

Mean Absolute Error (MAE), a statistical accuracy measure, is a commonly used measure of recommendation results. The smaller the MAE value, the better and the smaller it is, the higher the quality of the recommendation. Most experiments use MAE as a reference standard to measure the recommendation results. Let the set of predicted user ratings be denoted as {*p*_1_, *p*_2_,…, *p*_*n*_}, and the corresponding set of actual user ratings be {*q*_1_, *q*_2_,…, *q*_*n*_}; then, the mean absolute deviation MAE is defined as follows:

(12) $$\begin{array}{c} \text{MAE}=\frac{\sum_{i=1}^{N}\left|p_{i}-q_{i}\right|}{n}. \end{array}$$

#### 4.3.2. Decision Support Accuracy Criteria

Decision support accuracy criteria are used to evaluate the effectiveness of the prediction in assisting users to select the item that meets their interests from among many items. This type of criterion is based on the premise that the prediction process is viewed as a process in which the user makes a choice decision, i.e., the user thinks the item is good, or poor, or the user either chooses the item or does not choose the item. Based on the above assumptions, for evaluating items on a scale of 1–5, if the user chooses an item with a predicted value of 4 or higher, then there is no difference between a predicted value of 1 and 2. Commonly used criteria in this category are Reversal Rate, Weighted Errors, and ROC Sensitivity.

In this paper, the subject operating characteristic curve (ROC Sensitivity curve) and the area under the ROC curve (AUC) are chosen as measures to compare the quality of different method recommendations. The ROC curve reflects the system sensitivity, and the larger the area under the ROC curve is, the more accurate the recommendation system is.

### 4.4. Operation Program

The program computing environment is as follows:

  CPU: Core (TM) 2 Duo CPU 2.00 GHz
  Memory: 2.00 GB
  Hard disk: 250 GB
  Operating system: Microsoft Windows 7
  Tools and software: Microsoft Office Excel 2010 and Matlab 7.1
  The main considerations of this paper for the quiz include the following:

1. Comparison of the recommended effects of different methods when the data and variables are determined
2. The recommended effectiveness of all methods in the case of changing the number of ranks
3. Exploring whether there is a certain relationship between the choice of method and the choice of rank, and the degree of influence of both on the experimental recommendation results
4. Comparison of the recommendation effects of various methods in the case of different transaction (viewing) frequencies
5. Whether there are significant differences in the recommendation effects of various methods under the case of changes in the number of iterations
6. Comparison of the recommendation effects achieved by various methods as the item life cycle changes (within a certain range)

In the experiments, the various methods are labelled with English abbreviation codes for greater clarity and conciseness. The abbreviation codes for each type of method are shown in Table 2.

## 5. Experimental Results and Discussion

The contents to be achieved by the experiment include the following:

1. Experimental purpose: to compare the AUC values of various recommendation algorithms and to judge the strengths and weaknesses of the recommendation effects by the AUC curves.
2. Experimental parameters: training set: test set = 80%:20%; similarity measurement method is correlation similarity measurement.
3. Experimental method: the AUC curves derived from different methods are obtained by setting different variables to change, so as to judge the sensitivity of the data to the method.
4. Experimental inputs: user-item rating matrix, similarity measure, training set test set ratio, and so on.
5. Experimental output: AUC sensitivity curves.
6. Experimental results and analysis.

### 5.1. Firstly, the Singular Value Decomposition Is Performed on the Huge Matrix

The specific decomposition process is as follows:

 
*y* = textread (‘*u*1.base.text');
  [*m*, *n*] = size(*y*);
  training = zeros (1682, 943);
  for *i* = 1: *m*
  training (*y*(*i*, 2), *y*(*i*, 1) = *y*(*i*, 3));
  end
  [*U*, *S*, *V*] = svd (training)

After obtaining the singular values of the matrix, the matrix can be reconstructed to obtain a matrix of lower dimensionality for the following operations.

### 5.2. Comparison of the Recommendation Effects of Different Methods Obtained from One Experiment with Various Variables Determined

Let the user-item scores at each stage be subject to Poisson distribution and assume that the evaluation stage is 16 hours and the life cycle of the item is 4 weeks, and let its latent factor (rank) be 3 with an error range of 10^−8^.

**Figure 1:**
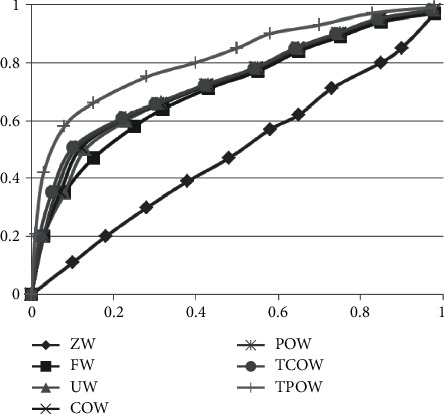
The comparison of various kinds of recommendation methods after a run.

The default values are brought in and then the above types of methods are validated according to the algorithmic process. The results are shown in Figure 1.

As can be seen in Figure 1, the 0-weighted method is the worst performer, and the subject work characteristic curve it presents, i.e., the ROC curve, exhibits almost a diagonal form and does not provide any categorical features. In contrast, in the other benchmark methods, the ROC curves are presented in a similar form, with some points having significantly higher values and higher perceptibility. For the methods studied in this paper, the intergroup effects item (movie)-centered weighting method has the best sensitivity performance, i.e., the closer the ROC curve is to the upper left corner, the higher the accuracy. The intergroup effects user-centric weighting method with slightly lower sensitivity also performs better than the other benchmark methods.

### 5.3. Consider the Effect of Different Rank Variations on Different Types of Methods to Generate Recommendations

To make the training results more accurate and the comparison of various methods more obvious, the number of iterations is increased so that the number of iterations is 10, and after running, the area under the ROC curve (AUC) presented by the various methods is compared.  Table 3 shows the mean and standard deviation for different rank cases.

**Figure 2:**
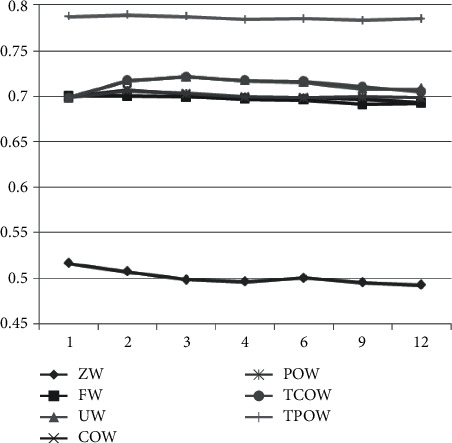
The mean change of different methods with different ranks.

Plotting the averages of the various methods in Table 3 into a graph can make the results more concise and clear as shown in Figure 2.

In Figure 2, the mean and variance of AUC of the 0-weighted (ZW) method are under 0.6; thus, this method is less likely to be recommended while being compared with the other methods. Among the remaining other methods, the intergroup effects product-centered weighting (TPOW) and the intergroup effects customer-centered weighting (TCOW) are both more effective than the Uniform Weighting (UW) method, the customer-centered weighting (COW) method, and the product-centered weighting (POW) method in terms of AUC performance. On the other hand, it can also be found from Figure 2 that as the rank increases, i.e., the number of potential features increases, the effect goes through a phase of first convergence to saturation and then degradation to decline. An analysis of variance (ANOVA) of the different methods and ranks allows further exploration of the statistical relationships and possible interactions between them.

A brief analysis shows that the *p* value of the rank indicated by the rows is smaller, while the *p* value of the method indicated by the columns is larger, so that the method has a greater degree of influence than the rank.

### 5.4. Influence of Different Transaction (Rating) Rates on the Recommendation of Different Methods

**Figure 3:**
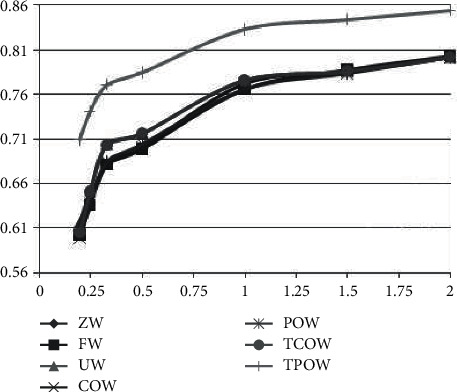
The comparison of different methods recommendation with different trading frequencies.

Considering different transaction (scoring) rates, the corresponding conversion rate can be calculated by taking two weeks as a time unit (10 days) and 16 hours in a day as a transaction (scoring) active period. For example, in Figure 3, 0.2 trades per minute would be equivalent to 2880 trades per period and 0.5 trades per minute would be equivalent to 7200 trades per period. When the trade rate increases, i.e., when the matrix of valid data increases, it is clear that all these methods have a good model learnability.

### 5.5. Comparison of the Recommended Effects of Various Methods at Different Assessment Stages

**Figure 4:**
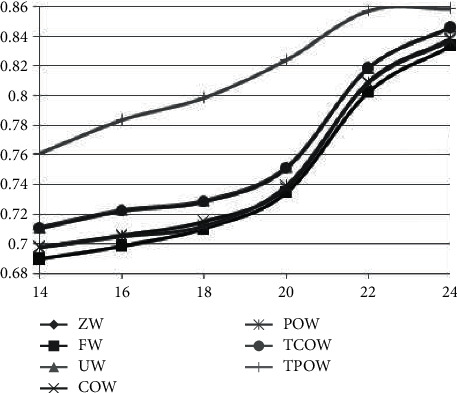
All kinds of recommendations comparison in different stages.

The evaluation of different methods in different stages also needs to be explored. In one experiment, for the evaluation recommendation of stage *N*, model learning needs to take into account the historical transaction (viewing) records of the previous (*N* − 1) stages. The performance effectiveness of all methods improves as the historical records increase; however, the differences between methods describe the robustness (stability) of the different methods.  Figure 4 shows that the recommendation effect gradually improves as the evaluation stage increases, but it can be concluded from the figure that the recommendation effect of the weighted method based on intergroup effects information is still better than other methods and has some stability.

### 5.6. Impact of Different Iterations and Item Life Cycle on Different Types of Recommendation Methods

**Figure 5:**
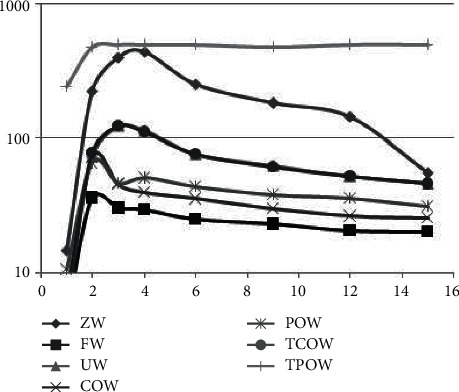
The comparison of different recommendations with different iterations.

In this paper, the experiments are run for a number of iterations less than 20, and a larger number of iterations are not considered (Figure 5). The intergroup effects item-centric weighting method performs best for the number of iterations less than 20, and the error gradually converges as the number of iterations increases.

**Figure 6:**
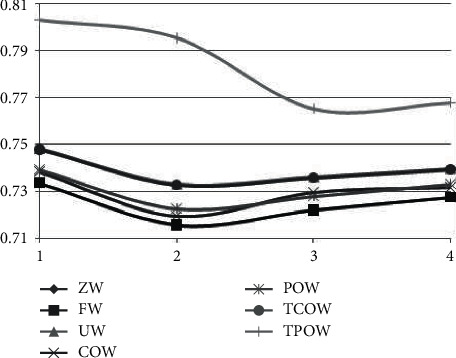
The comparison of different recommendations with different product life cycles.

Considering the life cycle PLC of the item, the change of the life cycle rate of the item is set. The shortest life cycle stage is 1 and the largest life cycle stage is 4, as detailed in Figure 6.

In this paper, ROC sensitivity curves are chosen to verify the recommendation quality of different methods, and both can conclude that the hidden semantic model based on intergroup effects information is better than other basic algorithms, and among the two algorithms based on intergroup effects information, the item-centered hidden semantic model based on intergroup effects information performs better. Overall, the following conclusions can be drawn from the experimental simulations in this paper:

1. The weighted method based on intergroup effects information is better than other basic methods at a certain identified evaluation stage, and on this basis, if the transaction frequency (i.e., evaluation stage information) and item life cycle are changed, the weighted method based on intergroup effects information still outperforms other weighted methods in general, while the item-centered weighted recommendation based on intergroup effects information is optimal and the recommendation effect of the 0-weighted method is the least desirable. It also illustrates, to a certain extent, the necessity and importance of introducing weight settings and supplementing negative examples for hidden semantic problems.
2. By exploring the recommendation effect of all methods with changing the number of ranks and the number of iterations, it is found that the effect of rank on the recommendation effect is not too obvious compared with the difference between methods, and the recommendation effect of each method gradually shows convergence as the number of ranks increases. In addition, with the increase of the number of experimental iterations, the recommendation effect of various methods also showed convergence, but in general, the weighting method based on intergroup effects information performed the most stable.

Therefore, personalized recommendation technology has been rapidly developed and applied to e-commerce recommendation systems, which can help users to avoid getting lost in the huge amount of item information, help them to make decisions, and select the desired items. With the help of the model, users can avoid getting lost in the huge amount of item information, help them to make decisions, select the desired items, and increase the sales volume of items; especially, in the arrival of “big data era,” information overload, and information explosion, the hidden semantic technology in personalized recommendation technology has gradually become the most mature and successful tool for users.

## 6. Conclusion

In the era of rapid development of e-commerce, a website with an excellent e-commerce recommendation system can not only gain the love of users-users but also help enterprises or merchants discover the useful information in users' information, so as to improve their marketing and promotion strategies. In addition to the popular recommendation technology, what is more important in e-commerce recommendation system is the personalized recommendation method. Good recommendation technology can make e-commerce websites achieve good personalized recommendation effect, and hidden semantic is the best among many technologies. In the field of hidden semantic, there is a relatively new research topics, namely, hidden semantic, whose treatment of negative examples can improve the recommendation effect to a great extent. The work done is summarized as follows:

1. A brief introduction is given on one area of hidden semantic, based on which the common underlying algorithms and the problems are analysed and explained.
2. A hidden semantic recommendation model based on intergroup effects information is developed by using different weighting methods and introducing intergroup effects information, such as users' historical visits (evaluation phase) and items' input market information (items' life cycle).
3. The designed hidden semantic model based on intergroup effects information was experimentally processed: Firstly, the data set was processed by applying the singular value decomposition matrix method to reduce the dimensionality of the huge matrix to a certain extent, and then the algorithms in this model were experimentally compared with the already existing basic methods. From the experimental results, it can be seen that the hidden semantic model based on intergroup effects information in this paper outperforms the other basic methods and obtains good results in the experiments conducted.

Of course, this paper also has research shortcomings. In this paper, we present a comprehensive solution to the problem of hidden semantic, which has many problems of its own, including sparsity and intergroup effects while the solution of this hidden semantic problem based on intergroup effects information will have positive implications for cross-selling, personalized, and targeted recommendation sales in the field of e-commerce.

## Figures and Tables

**Table 1:** A summary table on literature review.

Authors	Contributions	Gaps
Wang et al. [6], Hu et al. [7], Steck [8], Paterek [9], and Rendle et al. [10]	Treating all unselected or unrated items or items as negative examples and setting certain weights.	Compared with hidden semantic, there are fewer studies on one hidden semantic, and the sparse data, fewer positive cases, and more confusing negative cases make the research difficult, but it also shows that one hidden semantic has potential research value and space.
Chen et al. [11], Shi et al. [12], and Pan et al. [13]	A certain sample of unselected or unrated items or items is taken as negative examples.	
Pan and Scholz [5], Jiang et al., Sindhwani et al., Pan et al., Shen et al., Wang et al., Yin et al., Kang et al., Tian et al., and Shi et al., [14–22]	Certain rules are artificially set so as to specify negative examples, but this method especially relies on the special application environment as well as human a priori knowledge.	

**Table 2:** All kinds of methods and the corresponding English abbreviation code.

Method	Abbreviation code
0-weighted	ZW
Full weighted	FW
Uniform weighting	UW
Customer-centric weighting	COW
Product-centric weighting	POW
Intergroup effects customer-centric weighting	TCOW
Intergroup effects, product-centric weighting	TPOW

**Table 3:** The mean and standard deviation of different ranks.

	Ranks													
	1		2		3		4		6		9		12	
	*μ*	*σ*	*μ*	Σ	*μ*	*σ*	*μ*	Σ	*μ*	*σ*	*μ*	*σ*	*μ*	*σ*
ZW	0.516	0.037	0.507	0.004	0.498	0.007	0.496	0.008	0.500	0.007	0.495	0.007	0.492	0.01
FW	0.700	0.021	0.700	0.025	0.699	0.021	0.697	0.022	0.696	0.021	0.691	0.022	0.692	0.02
UW	0.698	0.022	0.717	0.022	0.721	0.021	0.717	0.021	0.715	0.021	0.708	0.022	0.708	0.021
COW	0.699	0.022	0.707	0.023	0.702	0.024	0.698	0.023	0.698	0.024	0.697	0.024	0.693	0.022
POW	0.698	0.021	0.706	0.024	0.703	0.022	0.699	0.022	0.698	0.024	0.699	0.024	0.698	0.022
TCOW	0.698	0.022	0.717	0.022	0.721	0.022	0.717	0.021	0.716	0.021	0.71	0.022	0.705	0.021
TPOW	0.787	0.015	0.789	0.017	0.787	0.017	0.784	0.016	0.785	0.016	0.783	0.016	0.785	0.015

## Data Availability

All data could be accessed upon request to the corresponding author.
